# Anatomical Preparations in Wax

**Published:** 1830-07-01

**Authors:** 


### ANATOMICAL PREPARATIONS IN WAX.

We deem it a tribute to merit, and a duty to the medical public, to notice and recommend the anatomical wax preparations executed by an ingenious German, (A. SCHLOSS, 50, Fore-street, Cripplegate) in a manner far superior to any thing of the kind which we have yet seen, and at a very moderate expense. We had lately opportunities of very minutely examining the wax-works in the museums of Bologna and of Florence, and we have no hesitation in giving the decided preference to the German manufacture. The Italians tell us, indeed, that their delightful climate conduces so much to the ductility of their *pure* wax, that the English need never hope to imitate, much less excel them. Be this as it may, GERMAN INDUSTRY and PATIENCE have, in this instance, as much overcome Italian genius, in the art of modelling, as the sturdy warriors from the banks of the Rhine formerly surpassed the effeminate Romans in the art of war. In a late meeting of the College of Physicians, several specimens of M. Schloss's works were there exhibited, and we may fearlessly appeal to the numerous judges there assembled, for the truth of this assertion. We, therefore, invite attention to, as the best recommendation of, Mr. Schloss's collection.

---

